# Livestock-Associated Methicillin Resistant and Methicillin Susceptible *Staphylococcus aureus* Sequence Type (CC)1 in European Farmed Animals: High Genetic Relatedness of Isolates from Italian Cattle Herds and Humans

**DOI:** 10.1371/journal.pone.0137143

**Published:** 2015-08-31

**Authors:** Patricia Alba, Fabiola Feltrin, Gessica Cordaro, María Concepción Porrero, Britta Kraushaar, María Angeles Argudín, Suvi Nykäsenoja, Monica Monaco, Marc Stegger, Frank M. Aarestrup, Patrick Butaye, Alessia Franco, Antonio Battisti

**Affiliations:** 1 Istituto Zooprofilattico Sperimentale del Lazio e della Toscana “M. Aleandri”, Diagnostic Department, Rome, Italy; 2 VISAVET Health Surveillance Centre, Universidad Complutense Madrid, Madrid, Spain; 3 Federal Institute for Risk Assessment (BfR), Berlin, Germany; 4 Veterinary and Agrochemical Research Centre (VAR-CODA-CERVA), Groeselenberg, Belgium; 5 Finnish Food Safety Authority Evira, Helsinki, Finland; 6 Istituto Superiore di Sanità, Department of Infectious, Parasitic and Immuno-mediated Diseases, Rome, Italy; 7 Statens Serum Institut, Department of Microbiology and Infection Control, Copenhagen, Denmark; 8 National Food Institute, Technical University of Denmark, Kgs. Lyngby, Denmark; 9 Ross University, School of Veterinary Medicine, Department of Biosciences, Basseterre, St. Kitts and Nevis, West Indies; 10 Ghent University, Faculty of Veterinary Medicine, Department of Pathology, Bacteriology and poultry diseases, Merelbeke, Belgium; 11 Université Libre de Bruxelles, Department of Microbiology, Hôpital Erasme Laboratoire de Référence MRSA—Staphylocoques, Brussels, Belgium; Institut National de la Recherche Agronomique, FRANCE

## Abstract

Methicillin-resistant *Staphylococcus aureus* (MRSA) Sequence Type (ST)1, Clonal Complex(CC)1, SCC*mec* V is one of the major Livestock-Associated (LA-) lineages in pig farming industry in Italy and is associated with pigs in other European countries. Recently, it has been increasingly detected in Italian dairy cattle herds. The aim of this study was to analyse the differences between ST1 MRSA and methicillin-susceptible *S*. *aureus* (MSSA) from cattle and pig herds in Italy and Europe and human isolates. Sixty-tree animal isolates from different holdings and 20 human isolates were characterized by pulsed-field gel electrophoresis (PFGE), *spa*-typing, SCC*mec* typing, and by micro-array analysis for several virulence, antimicrobial resistance, and strain/host-specific marker genes. Three major PFGE clusters were detected. The bovine isolates shared a high (≥90% to 100%) similarity with human isolates and carried the same SCC*mec* type IVa. They often showed genetic features typical of human adaptation or present in human-associated CC1: Immune evasion cluster (IEC) genes *sak* and *scn*, or *sea*; *sat* and *aphA*3-mediated aminoglycoside resistance. Contrary, typical markers of porcine origin in Italy and Spain, like *erm*(A) mediated macrolide-lincosamide-streptograminB, and of *vga*(A)-mediated pleuromutilin resistance were always absent in human and bovine isolates. Most of ST(CC)1 MRSA from dairy cattle were multidrug-resistant and contained virulence and immunomodulatory genes associated with full capability of colonizing humans. As such, these strains may represent a greater human hazard than the porcine strains. The zoonotic capacity of CC1 LA-MRSA from livestock must be taken seriously and measures should be implemented at farm-level to prevent spill-over.

## Introduction

Methicillin-resistant *Staphylococcus aureus* (MRSA) *spa*-type t127, Sequence Type (ST) 1, was first reported as one of the three most prevalent MRSA lineages in Italian pig industry, present in 6% of the holdings surveyed in Italy in 2008 [1]. ST1 belongs to Clonal Complex (CC)1, a particularly successful lineage associated with human infections, which includes Panton-Valentine (PVL)-positive CA-MRSA also known as USA400 [2]. In Europe, PVL-positive ST1 CA-MRSA *SCCmec* V, was firstly reported in a Dutch patient (2005–2006) [3], then in an Italian patient (2007) after travel in the USA [4], and in Denmark [5]. In the United Kingdom, PVL-negative ST1, *spa*-type t127, *SCCmec* IVa isolates are among the most common CA-MRSA, often associated with injecting drug-users and homeless people [6]. Furthermore, ST1 *spa*-type t127 isolates have been reported as the sixth most prevalent clone, both MSSA and MRSA, isolated from human invasive infections in Europe [7,8].

As previously reported [9] *spa*-type t127 ST1 isolates of porcine origin shared a 75% similarity when tested by pulse field gel electrophoresis (PFGE) with isolates of human origin. They showed mainly differences in the *SCCmec* cassette and in some virulence and antimicrobial resistance marker genes.

In Italy, in the last five years, the CC1 lineage has also been increasingly detected in dairy cattle mastitis (Battisti, unpublished) [10, 11], in bulk milk from dairy cattle, and occasionally from goat milk and colonizing veal calves and small ruminants [12]. The aim of the study was to analyse the differences between isolates from food-producing animals and humans isolated in Italy and other European countries and to determine their genetic relatedness for epidemiological and risk assessment purposes.

## Materials and Methods

**Fig 1:**
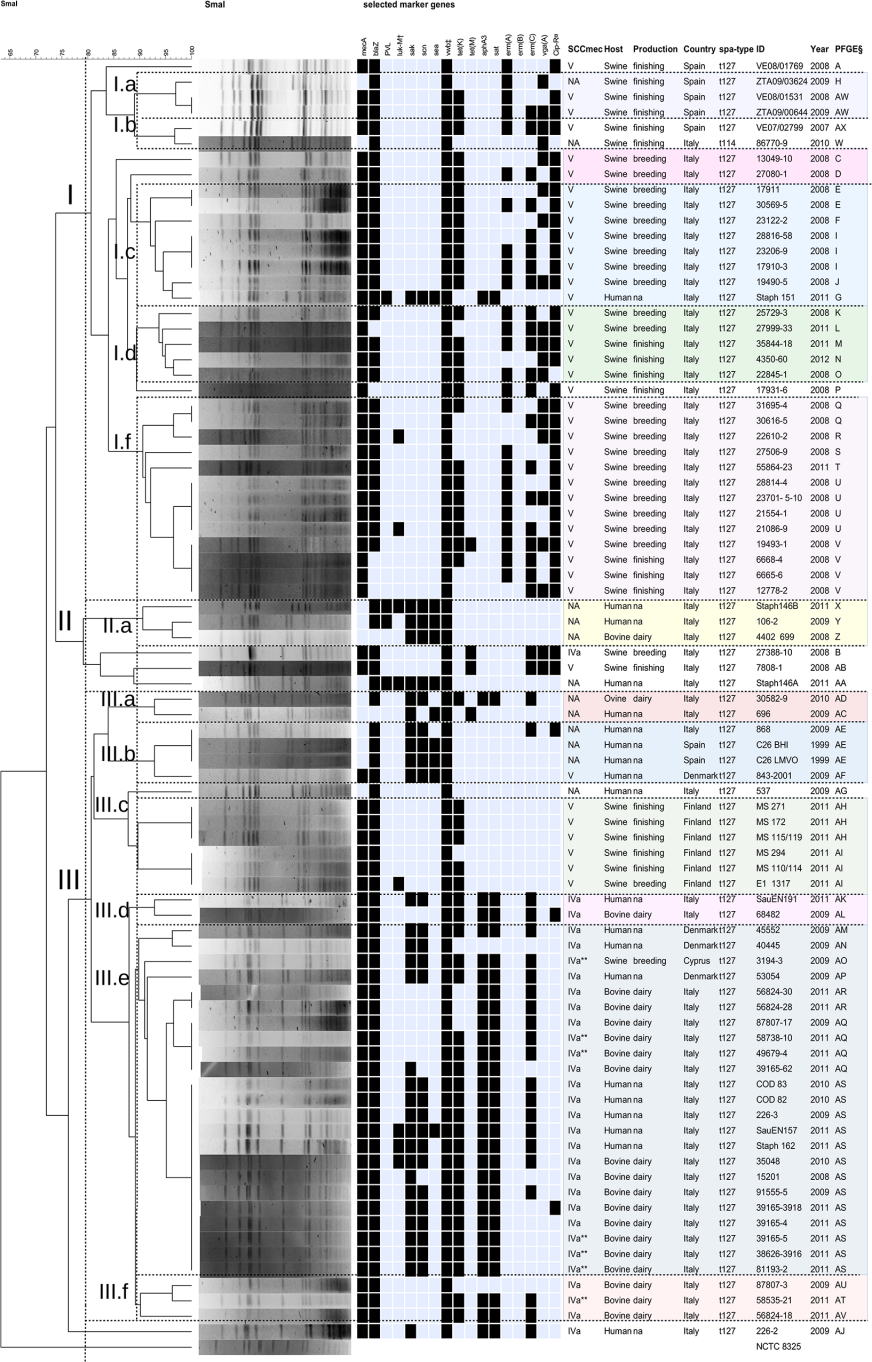
Dendrogram of SmaI PFGE macrorestriction patterns, with selected virulence and antimicrobial resistance marker genes and elements of Sequence Type 1, Clonal Complex 1 MSSA and MRSA from animals and humans. Legend: ^†^ LukF-PV(P83)-LukM; ^‡^ vwb (COL+MW2); ^☼^Ciprofloxacin resistance; ^§^ PFGE profile. Notes: *SCCmecV&*SCCfus*; ** *SCCmec* IV (2B&5), subtype IVa.

A total of 83 ST(CC)1 *S*. *aureus* (71 MRSA and 12 MSSA) were studied (Fig 1). The majority (n = 65) were from Italy (including 30 MRSA from pigs, 18 MRSA from bovines, 8 MRSA from humans; 1 MSSA from bovine, 1 MSSA from pig, 1 MSSA from ovine, and 6 MSSA from human) and the remainders from Finland (6 MRSA from pigs), Spain (4 MRSA and one MSSA from pigs and 2 MSSA from humans), Denmark (4 MRSA from humans), Cyprus (1 MRSA from pigs).

Isolates from animals (n = 63, 2007–2011) were collected from population-based studies in different primary productions (surveys in pigs, cattle), and passive laboratory surveillance programmes (mastitis in dairy cattle and sheep). The Italian isolates originated from farms located in Northern and Central Italy. For the purpose of the study, only one representative isolate per *spa*-type and ST per epidemiological unit (i. e. per holding) was included. The 20 isolates of human origin (8 MSSA and 12 MRSA, 1999–2011) investigated in this study were derived from previous laboratory surveillances [5,8,9,13], originated from infections, and were of unknown epidemiological relationships with animal farming [9]. Metadata of the isolates under study are included in S1 Table.

Most of isolates of animal origin (e. g. nasal swabs, milk) were cultured as previously described [1], with slight modifications. Selective enrichment cultures were plated on Oxacillin Resistance Screen Agar (ORSA, Oxoid, Basingstoke, UK). Suspect *S*. *aureus* colonies (denim blue colonies on ORSA) were subcultured in 5% sheep blood agar and incubated at 37°C for 24 h. In some cases, animal isolates from passive surveillance were detected by direct plating on 5% sheep blood agar or on selective agar (ORSA). *S*. *aureus* isolates were identified by means of standard techniques: colony morphology, Gram staining, catalase, coagulase tube test and further confirmed by PCR assay of the *nuc* gene as previously described [14].

In order to determine whether the difference between two proportions was significant (presence of genetic or phenotypic characteristics in isolates from swine *vs* isolates from ruminants and humans), the Fisher exact test was calculated by using the StatCalc utility of the Epi Info version 7.1.5 software (http://wwwn.cdc.gov/Epiinfo/7/index.htm).

### Antimicrobial Susceptibility Testing

Isolates were tested for their antimicrobial susceptibility by broth micro-dilution (Trek Diagnostic Systems, Westlake, OH, USA). MICs were interpreted according to the European Committee on Antimicrobial Susceptibility Testing (EUCAST; http://www.eucast.org), using epidemiological cut-offs. The following drugs were tested: penicillin, cefoxitin, ciprofloxacin, chloramphenicol, clindamycin, erythromycin, gentamicin, kanamycin, streptomycin, linezolid, quinopristin/dalfopristin, fusidic acid, mupirocin, rifampicin, tetracycline, tiamulin, sulfamethoxazole, trimethoprim and vancomycin.

### Methicillin Resistance

All isolates were tested by PCR for the *mecA gene* [15], or by means of a multiplex PCR for *mecA*, *mecC*, *spa* and PVL genes [16].

### Genotyping

All the isolates were submitted to molecular characterization using *spa*-typing [17] and Multilocus Sequence Typing (MLST) [18]. The *SCCmec* typing of MRSA isolates was obtained by using gene-specific PCRs. Briefly, all the isolates were tested for the cassette types I-VI, VIII and IX using two multiplex PCRs [19]. In case of negative result for the *mec* gene complex, a PCR [20] for the *mec* gene C1 (present in the VII and X *SCCmec* types) was performed. Identified SCC*mec* type IV variants were further subtyped as previously described [21].

### Micro-Array Testing

Micro-array testing for the detection of a variety of pathogenicity and virulence-associated genes, antimicrobial resistance genes and strain- or host-specific markers of *S*. *aureus*, including the accessory gene regulators (*agr*) genes and superantigenic-toxin encoding genes was performed by a genotyping kit (Alere GmbH, Germany), as previously described [9]. The results were interpreted according to the manufacturer’s specifications.

### Macrorestriction-PFGE

All the CC1 *S*. *aureus* isolates were sub-classified using macrorestriction-PFGE [22, with modifications]. After 4h incubation with 30U of SmaI (Thermo Fisher Scientific, Waltham, USA) at 30°C, the digested plugs were placed in the agarose gel. The run was performed in a CHEF-DRII (Bio-Rad Laboratories GmbH, Munich, Germany) system according a previously harmonized protocol [23]. Cluster analysis was performed using BioNumerics 7 software (Applied Maths, Sint-Martens-Latem, Belgium), and a dendrogram was built applying the Dice Similarity coefficient with an optimization and tolerance of 1.5% each, and clustering using Unweighted Pair Group Method with Arithmetic Mean (UPGMA). *S*. *aureus* NCTC 8325 was the size standard strain used.

## Results

### Genotyping and Array Typing

All CC1 isolates were ST1, *spa*-type t127, except one ST1 MSSA isolate from an Italian pig holdings (*spa*-type t114). All but two porcine MRSA (Italy, Cyprus, with *SCCmec* IVa) carried *SCCmec* cassette type V (5C2), while all bovine and human MRSA carried SCC*mec* cassette type IVa, except two human isolates which carried *SCCmec*V&*SCCfus*, harbouring *fus*C, gene mediating fusidic acid resistance.

None of isolates from livestock and only 20% (4/20) of the human MSSA/MRSA isolates were PVL-positive (all isolates from Italy). All isolates were positive for genes of other leukotoxin families: LukF-LukS, LukD-LukE, Luk-X-LukY. All isolates carried the haemolysins (or putative membrane proteins) *hl*, *hlb*, *and hlIII* genes and 90% (75/83) also carried the *hla* gene. All MRSA isolates form pigs presented the undisrupted variant of the *hlb* gene, except the isolate from Cyprus.

Nineteen out of twenty human isolates (12 MRSA and 7 MSSA), 10/19 bovine (9 MRSA and one MSSA), the ovine and the porcine isolates from Cyprus harboured IEC genes, *sak* and *scn*, except for two human isolates and two bovine isolates that tested positive for the *sak* gene only. All isolates belonged to the agr type III group. All isolates had a similar pattern of capsule and biofilm-associated genes, with *capH8; capI8; capJ8; capK8; icaA; icaC; icaD* as the most frequent pattern (66 MRSA and 9 MSSA (75/83)). All isolates carried genes encoding “microbial surface components recognizing adhesive matrix molecules” (MSCRAMMs): *bbp*, *clfA*, *clfB*, *cna*, *ebh*, *ebpS*, *eno*, *fib*, *fnbA*, *fnbB*, *map*, *sasG*, *sdrC*, *sdrD*, and all carried hyaluronate lyase genes (*hysA1*, *hysA2)*. Other virulence genes such as protease genes were also present in all isolates (the most frequent pattern was: *aur*; *splA*; *splB*; *splE*; *sspA*; *sspB*; *sspP*, found in 42/83 isolates). All isolates tested positive for the Von Willebrand binding protein gene *vwb* (COL+MW2 probe), and were negative for the SAPI-encoded *vwb* (RF122) allelic variant.

All isolates were positive for several superantigens/enterotoxin-like (*ssl*/*set*) genes, and for the *seh* [24]. 12/20 human isolates (5 MRSA and 7 MSSA) and one bovine isolate (MSSA) also carried *sea*, *seb*, *sek*, *seq* in different combinations, with none positive for *tst*1 (either human or bovine alleles) or any of the exfoliative toxin genes (*eta*, *etb* and *etd*).

As regards antimicrobial resistance genes, 73% (52/71) of MRSA isolates carried the *tet*(K) gene, and only three MRSA from swine and one of human origin (MSSA) carried *tet*(M). Just one of these, a swine MRSA, carried both *tet*(K) and *tet*(M) genes, while only 4 swine MRSA were tetracycline susceptible. Gentamicin-kanamycin resistance (MIC > 4 mg/L; > 64 mg/L, respectively) mediated by the *aacA-aphD* genes, was a feature of the porcine cluster (9/43, 20.9%), often in co-presence with *aadD*, while all kanamycin-resistant bovine/ovine and human isolates (30/40, 75%) carried *aphA3* and *sat* (conferring streptothricin resistance) only. Conversely, this latter combination of genes was significantly lower and rare in pig isolates (Fisher exact test p<0.0001), where it was found in 1/11 (9.1%) of kanamycin-resistant isolates. An exception were three human MRSA isolates: among these, the Danish isolate carried the *aacA-aphD* and *aadD* genes, whereas the Italian ones carried both *aacA-aphD* and *aphA3* and *sat* genes (Fig 1). Additionally, amphenicol resistance (a feature specific of the Italian isolates) was mediated by *cat*, or *fex*A and *cfr*, detected in 14% (6/43) of pig isolates. Macrolide-lincosamide (ML)-resistance was mediated most frequently by *erm*(C) (n = 27), mainly in Italian isolates, or by *erm*(A) (n = 7 isolates, from Italy and Spain), or by both genes (n = 16 isolates from Italy; n = 2 from Spain). No *erm*(A)-mediated ML-resistance was found in human or bovine/ovine isolates. In one pig isolate from Spain, the *lnu* gene, mediating lincosamide resistance was found in combination with *erm*(A).

Pleuromutilin microbiological resistance (tiamulin MIC>4 mg/L) was detected in 24/43 (56%) pig isolates, and was found to be mediated by *vga*(A) gene in 47% of these isolates (20/43; 18 MRSA and 2 MSSA, Fig 1), and in one case by the *cfr* methyltransferase gene, also mediating amphenicol, oxazolidinone and streptogramin A resistance (S1 Table).

Many isolates (36/83, 43.4%) showed a fluoroquinolone resistance phenotype, with all but one isolates being of swine origin (32/33) and one of bovine origin, with a ciprofloxacin (CIP) MIC range 8 - ≥16 mg/L. The remainder two isolates (bovine and human origin, respectively) had a CIP MIC of 2 mg/L. Again, proportions of both pleuromutilin and fluoroquinolone resistance in isolates of swine origin were significantly higher from those found in isolates of human/ruminant origin (Fisher exact test p<0.0001). All isolates carried *sdrM*, a chromosomally-encoded multidrug efflux pump, and five porcine and one human isolates the plasmid borne *qac*C, conferring resistance to quaternary ammonium compounds. The complete data set of genes investigated, including the microarray results, and antimicrobial resistance patterns is available in S1 Table.

### Analysis of PFGE Macro-Restriction Patterns

The dendrogram deduced from the PFGE macrorestriction profiles allowed a separation of 50 different PFGE profiles (A to AX) clustering into three main clusters (I-III) when using an 80% similarity cut-off, and each of them could be partitioned in subclusters (a, b, c,…), based on a 90% similarity (Fig 1).

Specifically, cluster I is composed of swine isolates only, carrying SCC*mec* V (Italian and Spanish origin), and only one isolate of human origin (Staph 151, MRSA) although this human isolate was the only one presenting characteristics typical of human-associated CC1 (*sak* and *scn* genes within the IEC).

Conversely, cluster III is mainly composed of isolates from humans and from cattle, characterized by SCC*mec* type IV (2B and its variant 2B&5), harbouring other human-associated CC1 genes, such as the IEC genes, and the *sat* and *aph*A3 resistance genes. They lacked the *erm*(A) mediated macrolide-lincosamide resistance. Subcluster IIIe, comprising the majority of human and bovine isolates, includes thirteen PFGE-indistinguishable isolates of human and bovine origin, sometimes also sharing the same pattern of resistance genes (Fig 1). The single MSSA isolate of ovine origin (Italy) harbouring human-associated marker genes showed a high similarity (Dice coefficient >90%) with one human MSSA from Italy by PFGE (subcluster III.a). All pig isolates from Finland grouped together in the subcluster III.c. The only MRSA of swine origin clustering with human and bovine isolates (III.e) is a Cypriot isolate (from dust swab) with other genetic features (*SCCmec* IVa; *aphA3*, *sat*; *sak*, *scn*) associated with human isolates.

## Discussion

This study represents the first wide molecular characterization of MRSA CC1 isolates from farm animals, and offers a further insight into relatedness and similarities with a set of CA-MRSA isolates from in humans.

Macrorestriction-PFGE clusters showed an impressive concordance with host of isolation and a series of other genetic markers (virulence, antimicrobial resistance) that are often carried by mobile genetic elements (MGEs), bacteriophages, and subject to loss or acquisition in relation to host adaptation process, useful for molecular epidemiology (Fig 1). Among these, *SCCmec* V is clearly a feature of porcine isolates, almost all in cluster I, while human and bovine MRSA isolates (cluster III) are characterized by *SCCmec* IVa. An exception is a single human MRSA SCC*mec* V (Staph 151), clustering with the porcine isolates, although also showing phage- or transposon- encoded genes typical of human adapted strains (Fig 1). Interestingly, we also report the finding of *SCCmec*V&*SCCfus* in two human isolates (from Italy and Denmark), an occurrence that has only been scarcely reported so far [25].

Striking is that swine MRSA isolates from Finland, show a high degree of similarity (subcluster III.c), and although with a *SCCmec* V, they seem more closely related to the human-bovine cluster (grouped in subcluster III.b) than the porcine isolates from Italy and Spain (cluster I). Conversely, they lacked all other acquired resistance genes, except for *tet*(K) and *blaZ* (Fig 1), and fluoroquinolone resistance, which is a typical feature of porcine multidrug-resistant MRSA CC1 isolates from Italy and from Spain. This is probably explained by a low antimicrobial usage in Finnish pig industry as shown in the ESVAC Reports [26]. Additionally, among the specific genetic markers of the Italian and Spanish multidrug-resistant MRSA of porcine origin is the presence of pleuromutilin resistance, mostly attributed to the *vga*(A) gene and also contributing to combined resistance to streptogramins A and lincosamides [27], in agreement to what has been previously reported in porcine MRSA [9]. Interestingly, resistance to quaternary ammonium compounds, commonly used in sanitization procedures in pig holdings [28] was another feature of Italian porcine isolates (cluster I), and was detected in one Danish human isolate only (cluster III).

Typically, all isolated studied including those from cattle/sheep, lack the SAPI-encoded bovine *vwb* gene, an important trait of host-specific pathogenicity, and this may be considered as one of the indications of a recent transfer of this lineage to food-producing animals, and especially to the ruminant hosts. As for the enterotoxin gene profile, *seh* appears to be constitutive of the ST(CC)1, irrespective of the host of origin, as previously observed [9]. Additionally, the pattern *sea*-*seh*-*sek*-*seq* is a feature typical of human isolates, since it has been found in only one IEC-positive MSSA isolate of animal origin, from a dairy cattle herd, clustering with human isolates (cluster II).

Most interestingly, the vast majority of isolates from dairy cattle (and sheep), shared a lot of genetic features usually found in typical human-associated CC1 clones. Amongst these are the immune evasion cluster (IEC) genes *sak* and *scn*, located on β-haemolysin-converting bacteriophages [29] and *sea*. Indeed, among the *sak*- or *scn*-positive isolates, ten were also positive for the enterotoxin *sea* gene, of which n = 9 isolated from humans (3 MRSA and 6 MSSA), while only one (MSSA) from dairy cattle. Similarly, almost all isolates from cattle and sheep often carried *sat* and *aph*A3 resistance genes associated with Tn5405-like elements, to be considered as further markers of a human origin [30,31]. Another characteristic of the human-bovine profile was the absence of *erm*(A) mediated macrolide-lincosamide resistance, and the rare occurrence of fluoroquinolone resistance, which is an epidemiological marker for Italian and Spanish porcine isolates.

All these features may be suggestive of a recent adaptation to the bovine (ruminant) host of human-associated CC1 strains, compared to the porcine cluster. A possible pathway of human-to-cattle exchange may be direct contact between farm workers and animals or indirect exposure through farm environment. Exposure of dairy animals is likely to result in colonization and even infection, since it is known that ST1 MRSA is capable of causing mastitis in cattle and mastitis/colonization in small ruminants [12]. A longitudinal study on a single case of chronic mastitis in a dairy cow [10], reported shedding over time, with immune response indicating acute infection, and neutrophils as the most represented cell population. Indeed, the first case of ST1 *spa*-type t127 *SCCmec* IVa mastitis in cattle was reported in Hungary in 2002, and showed PFGE-indistinguishable isolates amongst dairy cattle and one of the workers at the farm [32]. Unfortunately no further molecular typing was performed on these strains.

In conclusion, all MRSA CC1 isolates studied, irrespective of their host (animal or human) origin possess several virulence genes and resistance genes towards major classes of antimicrobials, and a variety of other pathogenicity factors. In this respect, MRSA in farm animals, either of bovine or swine origin, are to be considered a hazard for the community, and may represent a serious therapeutic challenge in case of invasive infections in humans.

Additionally, the finding that most of ST(CC)1 MRSA and MSSA associated with cattle in Italy, besides being multidrug-resistant and showing very high genetic relatedness (>90–100% PFGE similarity) to human isolates, also possess some of the virulence and immunomodulatory (e. g. IEC cluster) marker genes associated with full capability of colonizing and infecting humans, is of further concern. Since this clone has been demonstrated causing mastitis in cattle, with shedding of the pathogen over the lactation cycle, dairy farming may represent a source of exposure for the community also through unpasteurized milk or dairy products. With these genetic characteristics, spill-over from food-producing animals in high-prevalence geographic areas should be taken seriously. Prevention efforts should include actions to be taken at farm-level, aimed at minimizing exposures in the community and in categories directly related to farm animal industry.

## Supporting Information

S1 Table Dataset with metadata, genes investigated by microarray or PCR, and antimicrobial susceptibility tests and patterns of n = 83 *S*. *aureus* (MSSA, MRSA) ST(CC1) of animal and human origin. Legend: ^#^: Ambiguous result.
(XLS)

## References

[1] Battisti A, Franco A, Merialdi G, Hasman H, Iurescia M, Lorenzetti R, et al Heterogeneity among methicillin-resistant *Staphylococcus aureus* from Italian pig finishing holdings. Vet Microbiol 2010; 142:361–6. 10.1016/j.vetmic.2009.10.008 19914010

[2] McDougal LK, Steward CD, Killgore GE. Pulsed-field gel electrophoresis typing of oxacillin-resistant *Staphylococcus aureus* isolates from the United States: establishing a national database. J Clin Microbiol 2003; 41:5113–20. 14605147 10.1128/JCM.41.11.5113-5120.2003 PMC262524

[3] Deurenberg RH, Nulens E, Valvatne H, Sebastian S, Driessen C, Craeghs J, et al Cross-border dissemination of methicillin-resistant *Staphylococcus aureus*, Euregio Meuse-Rhin region. Emerg Infect Dis 2009; 15:727–734. 10.3201/eid1505.071618 19402958 PMC2687018

[4] Vignaroli C, Varaldo PE, Camporese A. Methicillin-Resistant *Staphylococcus aureus* USA400 Clone, Italy. Emerg Infect Dis 2009; 15:995–6. 10.3201/eid1506.081632 19523322 PMC2727323

[5] Larsen AR, Stegger M, Böcher S, Sørum M, Monnet DL, Skov RL. Emergence and characterization of community-associated methicillin-resistant *Staphyloccocus aureus* infections in Denmark, 1999 to 2006. J Clin Microbiol 2009; 47:73–8. 10.1128/JCM.01557-08 18971362 PMC2620878

[6] Otter JA, Havill NL, Boyce JM, French GL. Comparison of community-associated methicillin-resistant *Staphylococcus aureus* from teaching hospitals in London and the USA, 2004–2006: where is USA300 in the UK? Eur J Clin Microbiol Infect Dis 2009; 28:835–9. 10.1007/s10096-008-0698-9 19169720

[7] Grundmann H, Aanensen DM, van den Wijngaard CC, Spratt BG, Harmsen D, Friedrich AW. Geographic distribution of *Staphylococcus aureus* causing invasive infections in Europe: a molecular-epidemiological analysis. PLoS Med 2010; 7:e1000215 10.1371/journal.pmed.1000215 20084094 PMC2796391

[8] Monaco M, Pedroni P, Sanchini A, Bonomini A, Indelicato A, Pantosti A. Livestock-associated methicillin-resistant *Staphylococcus aureus* responsible for human colonization and infection in an area of Italy with high density of pig farming. BMC Infect Dis. 2013; 13:258 10.1186/1471-2334-13-258 23731504 PMC3679754

[9] Franco A, Hasman H, Iurescia M, Lorenzetti R, Stegger M, Pantosti A et al Molecular characterization of *spa* type t127, sequence type 1 methicillin-resistant *Staphylococcus aureus* from pigs. J Antimicrob Chemother 2011; 66:1231–5. 10.1093/jac/dkr115 21447518

[10] Pilla R, Castiglioni V, Gelain ME, Scanziani E, Lorenzi V, Anjum M, et al Long-term study of MRSA ST1, t127 mastitis in a dairy cow. Vet Rec 2012; 170:312. 10.1136/vr.100510 22383329

[11] Luini M, Cremonesi P, Magro G, Bianchini V, Minozzi G, Castiglioni B, et al Methicillin-resistant *Staphylococcus aureus* (MRSA) is associated with low within-herd prevalence of intra-mammary infections in dairy cows: Genotyping of isolates. Vet Microbiol. 2015; 178:270–4. 10.1016/j.vetmic.2015.05.010 26009302

[12] Cortimiglia C, Bianchini V, Franco A, Caprioli A, Battisti A, Colombo L, et al Prevalence of *Staphylococcus aureus* and methicillin-resistant S. aureus in bulk tank milk from dairy goat farms in Northern Italy. J Dairy Sci 2015; 98:2307–11. 10.3168/jds.2014-8923 25648812

[13] Argudín MA, Mendoza MC, Vázquez F, Guerra B, Rodicio MR. Molecular typing of *Staphylococcus aureus* bloodstream isolates from geriatric patients attending a long-term care Spanish hospital. J Med Microbiol. 2011; 60:172–9. 10.1099/jmm.0.021758-0 21030504

[14] Baron F, Cochet MF, Pellerin JL, Ben ZaKour N, Lebon A, Navarro A, et al Development of a PCR test to differentiate between *Staphylococcus aureus* and *Staphylococcus intermedius* . J Food Prot 2004; 67: 2302–2305. 15508648 10.4315/0362-028x-67.10.2302

[15] Strommenger B, Kettlitz C, Werner G, Witte W. Multiplex PCR Assay for Simultaneous Detection of Nine Clinically Relevant Antibiotic Resistance Genes in *Staphylococcus aureus* . J Clin Microbiol 2003; 41:4089–4094. 12958230 10.1128/JCM.41.9.4089-4094.2003 PMC193808

[16] Stegger M, Andersen PS, Kearns A, Pichon B, Holmes MA, Edwards G, et al Rapid detection, differentiation and typing of methicillin-resistant *Staphylococcus aureus* harbouring either mecA or the new *mecA* homologue mecA(LGA251). Clin Microbiol Infect 2012; 18:395–400. 10.1111/j.1469-0691.2011.03715.x 22429460

[17] Harmsen D, Claus H, Witte W, Rothgänger J, Claus H, Turnwald D, et al Typing of methicillin-resistant *Staphylococcus aureus* in a university hospital setting by using novel software for *spa* repeat determination and database management. J Clin Microbiol 2003; 41:5442–5448. 14662923 10.1128/JCM.41.12.5442-5448.2003 PMC309029

[18] Enright MC, Day NP, Davies CE, Peacock SJ, Spratt BG. Multilocus sequence typing for characterization of methicillin-resistant and methicillin-susceptible clones of *Staphylococcus aureus* . J Clin Microbiol 2000; 38:1008–1015. 10698988 10.1128/jcm.38.3.1008-1015.2000 PMC86325

[19] Kondo Y, Ito T, Ma XX, Watanabe S, Kreiswirth BN, Etienne J, et al Combination of multiplex PCRs for staphylococcal cassette chromosome mec type assignment: rapid identification system for mec, ccr, and Major Differences in Junkyard Regions. Antimicrob Agents Chemother 2007; 51: 264–274. 17043114 10.1128/AAC.00165-06 PMC1797693

[20] Katayama Y, Ito T, Hiramatsu K. Genetic organization of the chromosome region surrounding mecA in clinical staphylococcal strains: role of IS431-mediated mecI deletion in expression of resistance in mecA-carrying, low-level methicillin-resistant *Staphylococcus haemolyticus* . Antimicrob Agents Chemother 2001; 45:1955–63. 11408208 10.1128/AAC.45.7.1955-1963.2001 PMC90585

[21] Zhang K, McClure JA, Elsayed S, Louie T, Conly JM. Novel multiplex PCR assay for characterization and concomitant subtyping of staphylococcal cassette chromosome mec types I to V in methicillin-resistant *Staphylococcus aureus* . J Clin Microbiol 2005; 43:5026–5033. 16207957 10.1128/JCM.43.10.5026-5033.2005 PMC1248471

[22] Mulvey MR, Chui L, Ismail J, Louie L, Murphy C, Chang N, et al Development of a Canadian standardized protocol for subtyping methicillin-resistant *Staphylococcus aureus* using pulsed-field gel electrophoresis. J Clin Microbiol 2001; 39:3481–5. 11574559 10.1128/JCM.39.10.3481-3485.2001 PMC88375

[23] Murchan S, Kaufmann ME, Deplano A, de Ryck R, Struelens M, Zinn CE, et al Harmonization of pulsed-field gel electrophoresis protocols for epidemiological typing of strains of methicillin-resistant *Staphylococcus aureus*: a single approach developed by consensus in 10 European laboratories and its application for tracing the spread of related strains. J Clin Microbiol 2003; 41:1574–85. 12682148 10.1128/JCM.41.4.1574-1585.2003 PMC153895

[24] Su YC, Wong AC. Identification and purification of a new staphylococcal enterotoxin, H. Appl Environ Microbiol 1995; 61:1438–43. 7747964 10.1128/aem.61.4.1438-1443.1995 PMC167401

[25] Monecke S, Coombs G, Shore AC, Coleman DC, Akpaka P, Borg M, et al A field guide to pandemic, epidemic and sporadic clones of methicillin-resistant *Staphylococcus aureus* . PLoS One. 2011; 6:e17936 10.1371/journal.pone.0017936 21494333 PMC3071808

[26] European Medicines Agency, European Surveillance of Veterinary Antimicrobial Consumption, 2014. Sales of veterinary antimicrobial agents in 26 EU/EEA countries in 2012'. (EMA/333921/2014). Available: http://www.ema.europa.eu/docs/en_GB/document_library/Report/2014/10/WC500175671.pdf.

[27] Gentry DR, McCloskey L, Gwynn MN, Rittenhouse SF, Scangarella N, Shawar R, et al Genetic characterization of Vga ABC proteins conferring reduced susceptibility to pleuromutilins in *Staphylococcus aureus* . Antimicrob Agents Chemother 2008; 52:4507–9. 10.1128/AAC.00915-08 18838584 PMC2592886

[28] Merialdi G, Galletti E, Guazzetti S, Rosignoli C, Alborali G, Battisti A, et al Environmental methicillin-resistant *Staphylococcus aureus* contamination in pig herds in relation to the productive phase and application of cleaning and disinfection. Res Vet Sci 2013; 94: 425–7. 10.1016/j.rvsc.2012.10.020 23168262

[29] van Wamel WJ, Rooijakkers SH, Ruyken M, van Kessel KP, van Strijp JA. The innate immune modulators staphylococcal complement inhibitor and chemotaxis inhibitory protein of *Staphylococcus aureus* are located on beta-hemolysin-converting bacteriophages. J Bacteriol 2006; 188:1310–5. 16452413 10.1128/JB.188.4.1310-1315.2006 PMC1367213

[30] Derbise A, Dyke KG, Solh N. Characterization of a *Staphylococcus aureus* transposon, Tn5405, located within Tn5404 and carrying the aminoglycoside resistance genes, aphA-3 and aadE. Plasmid 1996; 35:174–88. 8812784 10.1006/plas.1996.0020

[31] Moon K, Shin C, Kim W, Im S. Linkage of kanamycin resistance gene with the streptothricin resistance gene in *Staphylococcus aureus* SA2. J Microbiol Biotechnol 1996; 6:219–220.

[32] Juhász-Kaszanyitzky E, Jánosi S, Somogyi P, Dán A, van der Graaf-van Bloois L, van Duijkeren E, et al, MRSA transmission between cows and humans. Emerg Infect Dis 2007; 13:630–632. 17553285 10.3201/eid1304.060833 PMC2725960

